# Artificial Intelligence Detection of Diabetic Retinopathy: Subgroup Comparison of the EyeArt System with Ophthalmologists’ Dilated Examinations

**DOI:** 10.1016/j.xops.2022.100228

**Published:** 2022-09-30

**Authors:** Jennifer Irene Lim, Carl D. Regillo, SriniVas R. Sadda, Eli Ipp, Malavika Bhaskaranand, Chaithanya Ramachandra, Kaushal Solanki

**Affiliations:** 1 Department of Ophthalmology, University of Illinois, Illinois Eye and Ear Infirmary, Chicago, Illinois; 2 Wills Eye Hospital, Thomas Jefferson University, Philadelphia, Pennsylvania; 3 Doheny Eye Center, Arcadia, California; 4 The Lundquist Institute, Harbor-University of California, Los Angeles Medical Center, Torrance, California; 5 Eyenuk, Inc., Los Angeles, California

**Keywords:** Artificial intelligence, Diabetic retinopathy, Screening, **AI**, artificial Intelligence, **CI**, confidence interval, **CSDME**, clinically significant diabetic macular edema, **DME**, diabetic macular edema, **DR**, diabetic retinopathy, **FDA**, Food and Drug Administration, **NPDR**, nonproliferative diabetic retinopathy, **mtmDr**, more than mild diabetic retinopathy, **PDR**, proliferative diabetic retinopathy, **vtDR**, vision-threatening diabetic retinopathy, **WFPRC**, Wisconsin Fundus Photograph Reading Center

## Abstract

**Objective:**

To compare general ophthalmologists, retina specialists, and the EyeArt Artificial Intelligence (AI) system to the clinical reference standard for detecting more than mild diabetic retinopathy (mtmDR).

**Design:**

Prospective, pivotal, multicenter trial conducted from April 2017 to May 2018.

**Participants:**

Participants were aged ≥ 18 years who had diabetes mellitus and underwent dilated ophthalmoscopy. A total of 521 of 893 participants met these criteria and completed the study protocol.

**Testing:**

Participants underwent 2-field fundus photography (macula centered, disc centered) for the EyeArt system, dilated ophthalmoscopy, and 4-widefield stereoscopic dilated fundus photography for reference standard grading.

**Main Outcome Measures:**

For mtmDR detection, sensitivity and specificity of EyeArt gradings of 2-field, fundus photographs and ophthalmoscopy grading versus a rigorous clinical reference standard comprising Reading Center grading of 4-widefield stereoscopic dilated fundus photographs using the ETDRS severity scale. The AI system provided automatic eye-level results regarding mtmDR.

**Results:**

Overall, 521 participants (999 eyes) at 10 centers underwent dilated ophthalmoscopy: 406 by nonretina and 115 by retina specialists. Reading Center graded 207 positive and 792 eyes negative for mtmDR. Of these 999 eyes, 26 eyes were ungradable by the EyeArt system, leaving 973 eyes with both EyeArt and Reading Center gradings. Retina specialists correctly identified 22 of 37 eyes as positive (sensitivity 59.5%) and 182 of 184 eyes as negative (specificity 98.9%) for mtmDR versus the EyeArt AI system that identified 36 of 37 as positive (sensitivity 97%) and 162 of 184 eyes as negative (specificity of 88%) for mtmDR. General ophthalmologists correctly identified 35 of 170 eyes as positive (sensitivity 20.6%) and 607 of 608 eyes as negative (specificity 99.8%) for mtmDR compared with the EyeArt AI system that identified 164 of 170 as positive (sensitivity 96.5%) and 525 of 608 eyes as negative (specificity 86%) for mtmDR.

**Conclusions:**

The AI system had a higher sensitivity for detecting mtmDR than either general ophthalmologists or retina specialists compared with the clinical reference standard. It can potentially serve as a low-cost point-of-care diabetic retinopathy detection tool and help address the diabetic eye screening burden.

Artificial intelligence (AI) systems using digital fundus photography instruments have been developed for diabetic retinopathy (DR) screening to partially address the increased demand for screening related to a burgeoning population with diabetes in the world. According to the International Diabetes Federation report of 2019, there are approximately 463 million persons with diabetes mellitus currently and this number is expected to increase to 700 million by 2045, an increase of almost 51%.^1^ In the United States, at the present time, best-case scenario estimates are that half of the patients with diabetes undergo screening.^2^^,^^3^ Screening for detection of DR is crucial because early treatment of DR is linked to better outcomes.^4^^,^^5^ Other options to address the screening burden include telemedicine screening programs, point-of-care screening, or traveling/mobile systems to provide access to patients.^6^ There are fixed costs associated with telescreening, which include the cost of individuals to obtain photographs, costs to transmit photographs to expert readers, and the time of the specialist to read the images. The use of traveling screening mobile units incurs an additional cost of transportation.

Advantages of AI screening systems include convenience of point-of-care access and the potentially lower operating cost as a result of automatic interpretation of the images and referral to an eye care specialist only as needed. The drawbacks of current telescreening and AI systems include the lack of an ability to detect and correct refractive errors, and lack of evaluation of the anterior segment and peripheral retina as would be performed during an in-person examination.

Two fully autonomous AI systems for detecting DR without human oversight have been cleared by the US Food and Drug Administration (FDA). The IDx-DR system is an FDA-cleared AI point-of-care screening system with 87% sensitivity, 90% specificity, and 96% imageability for more than mild DR (mtmDR).^7^ The EyeArt system (Eyenuk, Inc) is also an FDA-cleared AI-based system that can enable point-of-care screening with 96% sensitivity, 88% specificity, and 97% imageability for detecting eyes with mtmDR.^8^ Unlike IDx-DR, the EyeArt system can also detect eyes with vision-threatening diabetic retinopathy (vtDR) with 97% sensitivity and 90% specificity and provides DR detection results at the eye level.^8^ Moreover, to generate disease detection results, the EyeArt system requires fewer (12.6%) patients to be dilated than those required by the IDx-DR (23.6%).

The EyeArt system was developed using deep learning and uses multiple deep neural networks for specific classification tasks on images; the outcomes of these various networks are combined in a clinically aligned framework. It was trained on 375 000 images and then validated on 250 000 images.^9^ The AI analysis of the EyeArt system is cloud based with a user interface that is installed on the user’s computer. The system requires two 45° field of view images, 1 centered on the optic disc, and 1 centered on the macula, captured using a digital fundus camera. The images may be taken without dilation. The patient’s fundus photographs are uploaded to the cloud and are interpreted using the AI system within 60 seconds for the determination of mtmDR and vtDR. More than mild DR is defined as the presence of moderate nonproliferative DR (NPDR) or higher stage or the presence of diabetic macular edema (DME), and vtDR is defined as the presence of severe NPDR or proliferative DR (PDR) or the presence of DME. The patient disposition report generated for each patient visit contains mtmDR and vtDR determination for each eye. This report gives the follow-up recommendation as either referral to an ophthalmologist or return for screening in another year. The system is intended as a screening method to detect mtmDR and vtDR in eyes which have no prior treatment for DR.

In a prospective, pivotal study at 15 sites (primary care clinics, ophthalmology, and retina practices), the EyeArt system was shown to have high sensitivity and specificity to enable point-of-care screening.^8^ In this pivotal study, the clinical reference standard was standardized, adjudicated grading of stereoscopic 4 field, 45° digital fundus photographs^8^ on the ETDRS scale,^10^ as assessed by the Wisconsin Reading Center. The ability of the EyeArt system to detect the presence of mtmDR was compared with the Reading Center determination. The study showed that the AI system has a sensitivity of 96%, specificity of 88%, and gradability of 87.4% without dilation, increasing to 97.4% with dilation as needed. The EyeArt AI system favorably compared with the clinical reference standard of 4-field stereoscopic images and met the predetermined sensitivity and specificity end points for the detection of referable DR in individuals with diabetes (*P* < 0.0001), thus making the system suitable for point-of-care DR screening for triage and identification of patients requiring referral.

The purpose of this study was to compare the general ophthalmologists, retina specialists, and the EyeArt AI screening system with the clinical reference standard of Reading Center evaluation of fundus photographs for detection of mtmDR. Previous studies have compared dilated ophthalmoscopy with the ETDRS reference standard,^11^ AI systems with the ETDRS reference standard,^7^^,^^8^ AI systems with expert grading of nonstereo images,^9^^,^^12^ and AI systems with dilated ophthalmoscopy.^13^ To the best of our knowledge, this is the first study that evaluates and compares the performances of an AI system and dilated ophthalmoscopy with the ETDRS reference standard on the same large and diverse cohort of subjects enrolled at multiple centers with geographic diversity. Moreover, it also separately reports and compares the performance of dilated ophthalmoscopy by general ophthalmologists and retina specialists. Given that dilated ophthalmoscopy is the current standard of care for DR screening and that AI systems are alternatives that expand the reach of DR screening to primary care centers, this study comparing the 2 has significant clinical importance and impact.

## Methods

This analysis focuses on the subset of data from 10 of the 15 sites in the pivotal study (registered and publicly available at ClinicalTrials.gov, Identifier NCT03112005) where, a general ophthalmologist (i.e., nonretina practices) or a retina specialist (i.e., retina practices) participated in the trial to perform dilated eye examinations. The other 5 sites did not have a participating general ophthalmologist or a retina specialist who could perform dilated eye examinations. The exclusion of subjects enrolled at these 5 sites because of a lack of dilated ophthalmoscopy results does not introduce selection bias and does not alter the conclusions of this study. Approval from the University of Illinois at Chicago instituional review board was obtained and the study was conducted in adherence to the tenets of the Declaration of Helsinki. Following institutional review board approval and informed consent, patients were deemed eligible if they were diagnosed with diabetes mellitus, aged ≥ 18 years, and could tolerate fundus photography. Patients were ineligible if they had persistent visual impairment (defined by the American Foundation for the Blind as legal blindness or low vision where a person has difficulty accomplishing or cannot accomplish visual tasks even with prescribed corrective lenses^13^), history of macular edema, treatment for any form of DR (intravitreal injections, laser photocoagulation, and intraocular surgery other than for cataracts), or known retinal disease (vascular occlusion and retinal detachment). The enrollment criteria did not require knowledge of the patient’s ophthalmic history, including DR diagnosis.

Study participants underwent nonmydriatic imaging using the EyeArt system, followed by dilation and both dilated ophthalmoscopy and fundus photography. Trained ophthalmic photographers obtained two 45°, nonmydriatic color fundus digital images of the right eye followed by the left eye using a digital fundus camera with a resolution of ≥ 1.69 megapixels (Canon CR-2 AF or CR-2 Plus AF). For each eye, 1 image was centered on the optic disc and the other was centered on the macula. These nonmydriatic fundus photographs were uploaded to the cloud, where the EyeArt AI system determined whether mtmDR and vtDR (as defined earlier) were present. If the image quality precluded an interpretation of level of DR by the AI system, operators received immediate feedback from the EyeArt system, and the eye underwent dilation followed by repeat imaging and was uploaded for automated analysis. Otherwise, after the completion of nonmydriatic photographs and dilation, the study participant proceeded to the remainder of the study procedures.

Once pupillary dilation was achieved, the participant underwent dilated ophthalmoscopy (slit lamp and indirect ophthalmoscopy) by an ophthalmologist (general or retina specialist), who graded each eye for both the presence or absence of clinically significant DME (CSDME) as per the ETDRS^10^ and presence of any DR, NPDR (graded as mild, moderate, or severe), or PDR. The ophthalmologist had access to the participant’s ophthalmic history when available. Four mydriatic 45° field of view stereoscopic images (nasal retina, including optic disc, centered on macula, supratemporal to disc, and inferotemporal to disc) were then taken of each eye. These four 45° field of view images are equivalent to the 7-field ETDRS 30° images.^14^ Images were sent to the Wisconsin Fundus Photograph Reading Center (WFPRC) for ETDRS grading of the presence or absence of DME and ETDRS-level grading of the DR.^10^ At the WFPRC, 2 independent certified graders masked to the EyeArt results examined the images using standardized procedures^10^ to establish the clinical reference standard. Between graders, differences exceeding prespecified criteria were adjudicated by a third, more senior grader.

For ophthalmoscopy performance analyses, eyes were included if: (1) the color fundus photographs were evaluable by the Reading Center; (2) a dilated examination with a DR grading was performed by an ophthalmologist; and (3) an EyeArt analysis was obtained. For EyeArt performance analyses, eyes that were ungradable per the EyeArt system were considered equivalent to mtmDR positive because in regular clinical use of the EyeArt system, patients with mtmDR positive results or ungradable results would be referred to an eye care provider for further evaluation. The WFPRC gradings of the 4-widefield mydriatic photographs were used as the clinical reference standard for the determination of sensitivity and specificity when comparing ophthalmoscopy and the AI EyeArt DR gradings. False-negative rates were determined for each method.

Sensitivity was defined as the accuracy among positive detections, calculated as the ratio of positive tests to reference standard positives. Specificity was defined as the accuracy among negative detections, calculated as the ratio of negative tests to reference standard negatives. Imageability/gradability was defined as the percentage of eyes that received a disease detection result (positive or negative) among all eyes determined as gradable by the WFPRC. For each of these performance measures, 95% confidence interval (CI) calculations were performed to account for the correlation between the eyes of the same patient using methods presented by Kang and Lee.^15^

## Results

Of the 893 participants who enrolled in the clinical trial and completed the study procedures, 521 participants also underwent dilated ophthalmoscopy. Of the 521 participants, 221 eyes (from 115 participants) evaluated at retina practices and 778 eyes (from 405 participants) evaluated at general ophthalmology (nonretina) practices had evaluable clinical reference standard photographs and 43 eyes (including 2 eyes from 1 participant) did not have evaluable clinical reference standard photographs. One participant’s clinical reference standard images for each eye were deemed not evaluable by the Reading Center and therefore this participant could not be included in the analyses, which require comparisons to the reference standard images.

The demographic characteristics of these subjects are presented in Table 1. Of these 999 total eyes, 792 were read by the Reading Center as mtmDR negative and 207 were mtmDR positive. Of these 999 eyes, 26 were ungradable by the EyeArt system, leaving 973 that had both EyeArt and Reading Center gradings. The Reading Center gradings are shown in Table 2. Of the 973 eyes, 669 had no apparent DR, 108 had mild NPDR, 183 had moderate NPDR, 1 had severe NPDR, 11 had PDR, and 1 had questionable DR (ETDRS level 14 or 15) per the Reading Center gradings. There were therefore a total of 196 eyes with mtmDR.

**Table 1:** Demographic Characteristics for Subjects with Dilated Ophthalmoscopy Examinations at Nonretina Sites (N = 406), Retina Sites (N = 115), and Total (N = 521)

Subgroup		Total n/N (%)		Nonretina sites n/N (%)		Retina sites n/N (%)	
Age	< 65 yrs	370/521	(71.0)	291/406	(71.7)	79/115	(68.7)
	≥ 65 yrs	151/521	(29.0)	115/406	(28.3)	36/115	(31.3)
Gender	Male	244/521	(46.8)	176/406	(43.3)	68/115	(59.1)
	Female	277/521	(53.2)	230/406	(56.7)	47/115	(40.9)
Ethnicity∗	Hispanic/Latino	169/521	(32.4)	149/406	(36.7)	20/115	(17.4)
	Non-Hispanic/Latino	352/521	(67.6)	257/406	(63.3)	95/115	(82.6)
Race∗	American Indian or Alaska Native	2/521	(0.4)	2/406	(0.5)	0/115	(0.0)
	Asian	18/521	(3.5)	14/406	(3.4)	4/115	(3.5)
	Black or African American	105/521	(20.2)	58/406	(14.3)	47/115	(40.9)
	Native Hawaiian or other Pacific Islander	3/521	(0.6)	3/406	(0.7)	0/115	(0.0)
	Other	44/521	(8.4)	24/406	(5.9)	20/115	(17.4)
	White	349/521	(67.0)	305/406	(75.1)	44/115	(38.3)

∗ Race and ethnicity were self-reported.

**Table 2:** International Clinical Diabetic Retinopathy Severity Scale and CSDME Levels of 973 Analyzable Eyes (from STARD Chart in Fig 1) of Participants with Dilated Ophthalmoscopy

DR level CSDME	No Apparent DR	Mild NPDR	Moderate NPDR	Severe NPDR	PDR	Questionable	Total
Absent	669	108	122∗	1∗	8∗	0	908
Present	0	0∗	51∗	0∗	2∗	1∗	54
Questionable	0	0	10∗	0∗	1∗	0	11
Total	669	108	183	1	11	1	973

CSDME = clinically significant diabetic macular edema; DR = diabetic retinopathy; NPDR = nonproliferative diabetic retinopathy; PDR = proliferative diabetic retinopathy; STARD = Standards for Reporting of Diagnostic Accuracy Studies.

∗ Cases that are considered more than mild diabetic retinopathy positive per the Reading Center.

**Figure 1:**
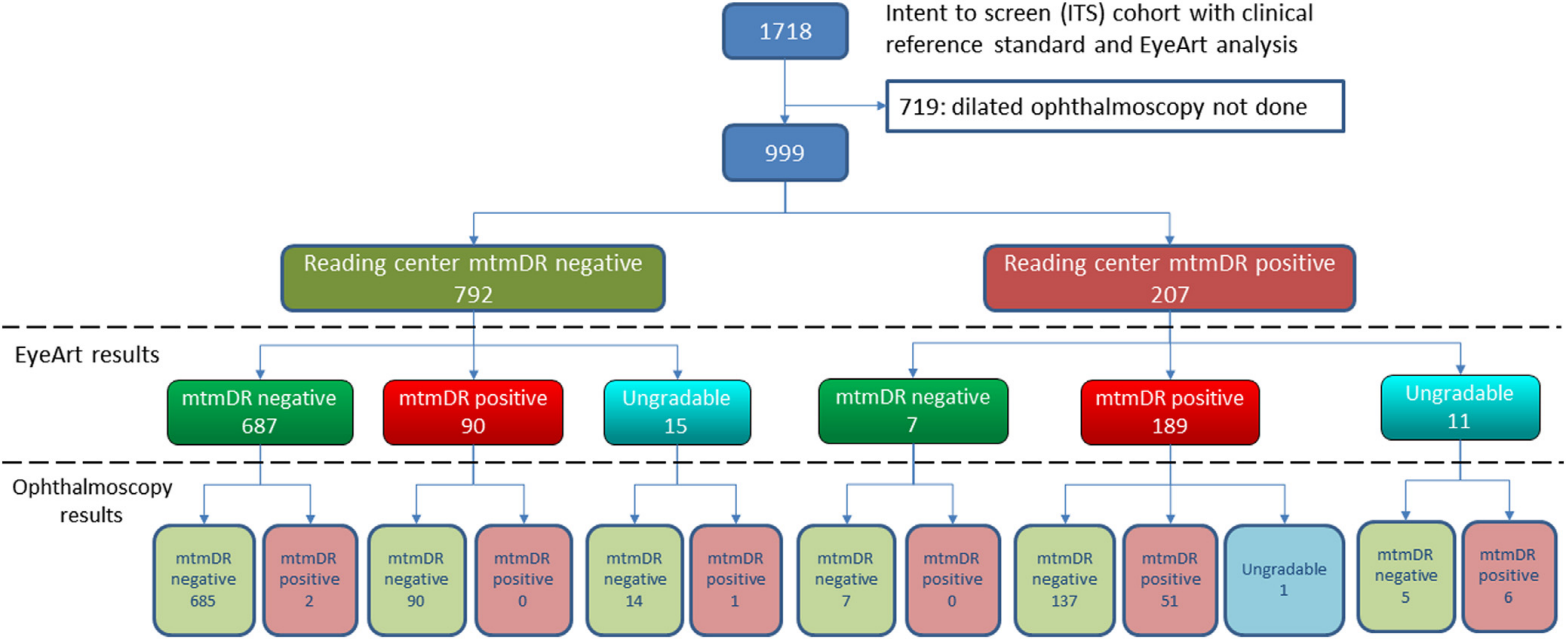
STARD chart of participants with dilated ophthalmoscopy (n = 999 eyes) showing the number of eyes that were positive, negative, or ungradable for more than mild diabetic retinopathy (mtmDR) per grading by the Reading Center, EyeArt system, and ophthalmoscopy by general ophthalmologists and retina specialists. STARD = Standards for Reporting of Diagnostic Accuracy Studies.

EyeArt and dilated ophthalmologist examination gradings were compared with Reading Center gradings. The breakdown of the EyeArt and dilated ophthalmoscopy results is shown in Figure 1 and the sensitivity, specificity, and imageability/gradability are reported in Table 3.

**Table 3:** Performance Characteristics (Sensitivity, Specificity, and Imageability/Gradability) for Subject Eyes with Dilated Ophthalmoscopy Examinations at Nonretina Sites (N = 778), Retina Sites (N = 221), and Total (N = 999)

Cohort (No. of Eyes with CRS)	Screening Methodology	Sensitivity (95% CI)	Specificity (95% CI)	Imageability/Gradability (95% CI)
Subjects with dilated ophthalmoscopy (n = 999 eyes)	Dilated ophthalmoscopy	27.7% [20.1%–35.2%]	99.6% [99.1%– 100.0%]	99.9% [99.7%–100.0%]
	EyeArt system (with dilation-if-needed) (147 eyes dilated)	96.4% [93.1%–99.8%]	88.4% [85.8%–91.1%]	97.4% [96.0%–98.8%]
Subjects enrolled at nonretina specialty centers (n = 778 eyes)	Dilated ophthalmoscopy	20.6% [13.1%–28.0%]	99.8% [99.5%–100.0%]	99.9% [99.6%–100.0%]
	EyeArt system (with dilation-if-needed) (109 eyes dilated)	96.5% [92.9%–100.0%]	86.3% [83.0%–89.7%]	97.6% [96.0%–99.1%]
Subjects enrolled at retina specialty centers (n = 221 eyes)	Dilated ophthalmoscopy	59.5% [40.2%–78.7%]	98.9% [97.1%– 100.0%]	100.0% [100.0%–100.0%]
	EyeArt system (with dilation-if-needed) (38 eyes dilated)	97.3% [90.3%–100.0%]	88.0% [82.5%–93.5%]	96.8% [93.6%–100.0%]

CI = confidence interval; CRS = clinical reference standard (as per reading center).

Overall, sensitivity for detection of mtmDR was higher for the EyeArt system than for dilated ophthalmoscopy: 96.4% (95% CI, 93.1%–99.8%) EyeArt versus 27.7% (95% CI, 20.1%–35.2%) for ophthalmoscopy. Specificity was lower for the EyeArt system than that for ophthalmoscopy: 88.4% (95% CI, 85.8%–91.1%) EyeArt versus 99.6% (95% CI, 99.1%–100.0%) dilated ophthalmoscopy.

Evaluation of the dilated ophthalmoscopy results showed that the retina specialists have higher sensitivity than the general ophthalmologists for the detection of mtmDR: 59.5% sensitivity retina specialists versus 20.7% for general ophthalmologists. The incidental demographic differences among the participants seen by the retina specialists and general ophthalmologists may have an impact on the performance differences in these subcohorts. Retina specialists correctly identified 22 of 37 eyes positive for mtmDR, resulting in a sensitivity of 59.5% and 182 of 184 eyes negative for mtmDR, resulting in a specificity of 98.9%. For this cohort, the EyeArt AI system correctly referred 36 of 37 eyes, resulting in a sensitivity of 97% and correctly identified 162 of 184 eyes as negative for mtmDR, resulting in a specificity of 88%. General ophthalmologists correctly identified 35 of 170 eyes as positive for mtmDR, resulting in sensitivity of a 20.6%, and correctly identified 607 of 608 eyes as negative for mtmDR, resulting in a specificity of 99.8%. For this cohort, the EyeArt AI system correctly referred 164 of 170 eyes, resulting in a sensitivity of 96.5% and correctly identified 525 of 608 eyes as negative for mtmDR, resulting in a specificity of 86%.

**Figure 2:**
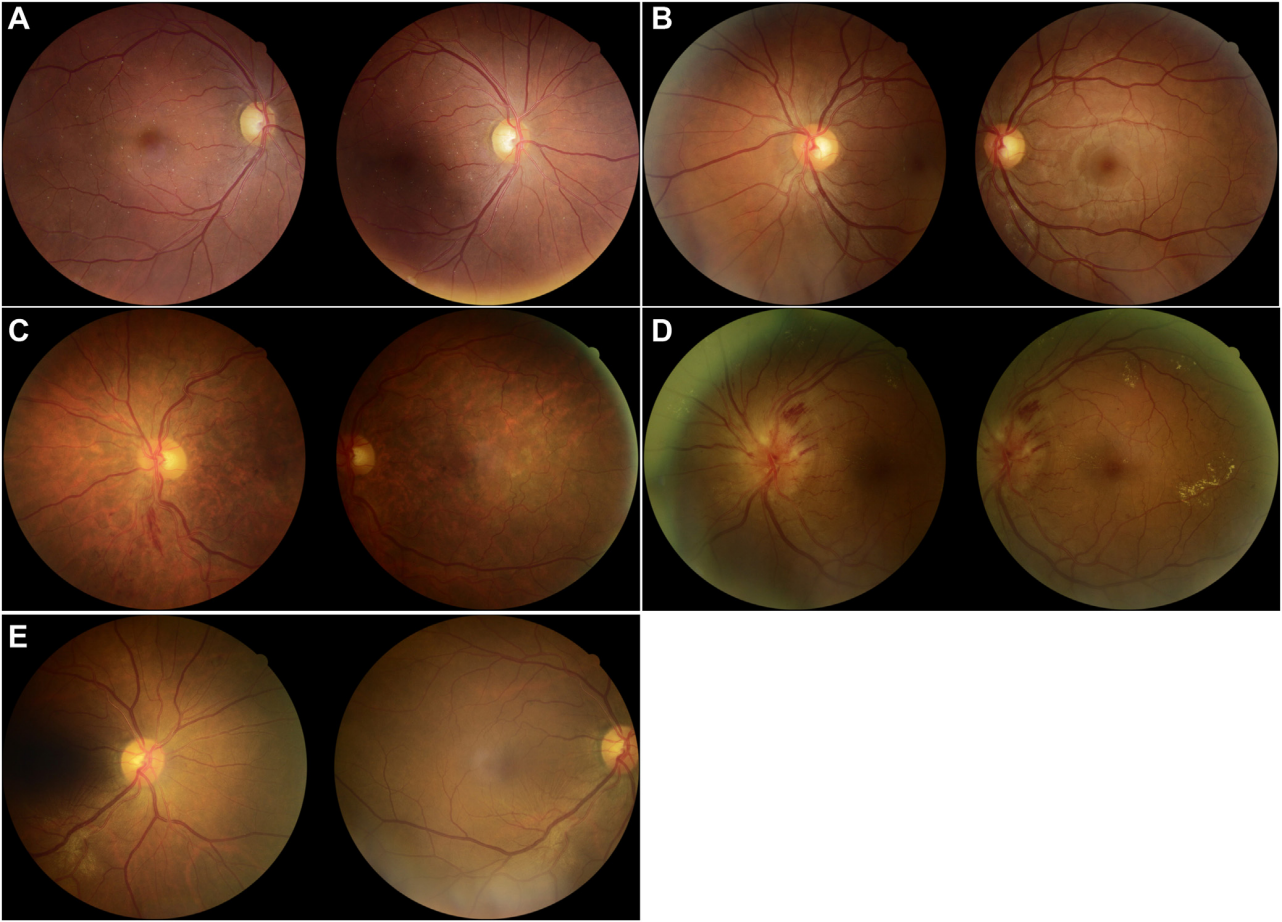
Two-field (EyeArt) images for eyes that were read as false-positive or false-negative per the EyeArt system compared with the grading by the Reading Center. **A**, EyeArt 2-field images read as negative for more than mild diabetic retinopathy (mtmDR) whereas the Reading Center 4-widefield images were graded as ETDRS 35C. No hemorrhages are visible on the images shown. **B**, EyeArt 2-field images read as negative for mtmDR whereas the Reading Center 4-widefield images were graded ETDRS 35C. Note there is a small hemorrhage just inferior to the superior arcade arteriole. **C**, EyeArt 2-field images read as vision-threatening diabetic retinopathy (vtDR) whereas the Reading Center diagnosed central vein occlusion. **D**, EyeArt 2-field images read as vtDR, whereas the Reading Center diagnosed swollen optic discs of unknown cause. **E**, EyeArt 2-field images read as mtmDR, whereas the Reading Center diagnosed an epiretinal membrane. Note the striae present in the macular area.

**Figure 3:**
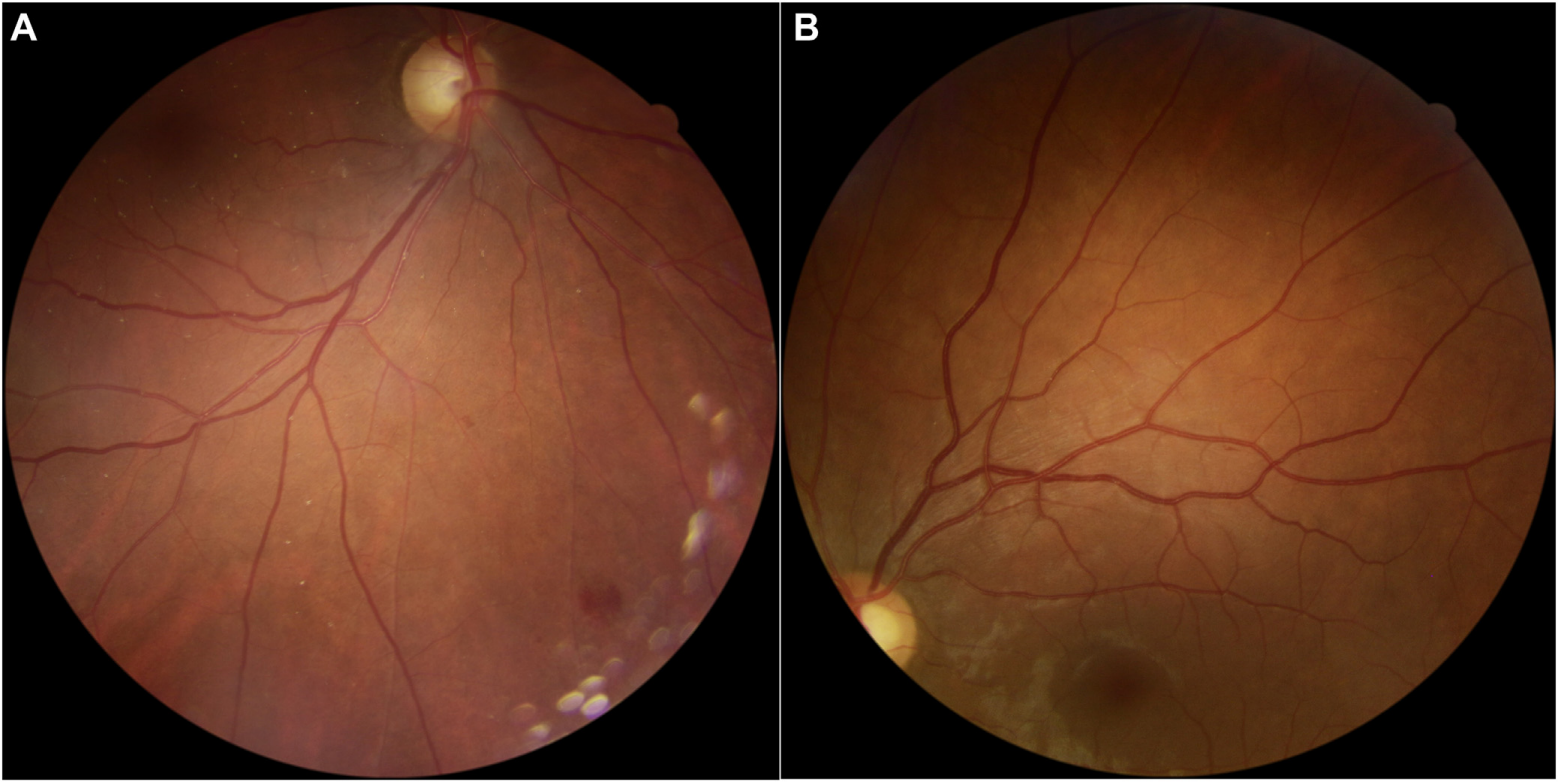
Color widefield 45**°** photographs corresponding to the representative 2-field EyeArt false-negatives for more than mild diabetic retinopathy (mtmDR) shown in Figure 2. **A**, This inferior field is the only 1 of the 4 widefield images that revealed any diabetic retinopathy. This image shows the presence of 2 small retinal hemorrhages and 1 larger hemorrhage. The larger hemorrhage was not in an area imaged by the EyeArt system. **B**, This widefield photograph reveals a small hemorrhage located just inferior to the superior arcade artery. This hemorrhage was visible on the 2-field EyeArt image but was read falsely negative for mtmDR. The other 3 widefield images of this eye did not show any retinopathy.

Among the 207 reference standard mtmDR positives, there were 18 false-negatives with the EyeArt AI system. Of these 18 eyes that were not identified as mtmDR positive by the EyeArt AI system, 7 read as negative and 11 as ungradable. All 7 reference standard positives read as negative by EyeArt were graded by the Reading Center as ETDRS level 35 without CSDME (Fig 2A, B). The corresponding widefield photographs show retinal hemorrhages that were not detected by the EyeArt images (Fig 3). In each case, only 1 of the 4 widefield images was positive for DR. The larger hemorrhage (Fig 3A) was in a retinal location beyond the 2-field area imaged by the EyeArt system.

Of the 11 reference standard positives read as ungradable by EyeArt, 8 eyes were read by the Reading Center as ETDRS level 35 (12 eyes), 2 as level 43, and 1 as level 47; none had CSDME. Thus, none were positive for vtDR as per the Reading Center. There were 149 false-negatives as read by the ophthalmologists. Of these 149 false-negatives, 15 of 149 (10%) were evaluated by retina specialists and the remainder (134 of 149, 90%) by general ophthalmologists. None of these retina specialists' false-negatives were read by the Reading Center as vtDR. In contrast, for general ophthalmologists, 26 of 134 (19%) eyes had vtDR. These included 5 eyes with PDR, 7 with level 47 and CSDME, 3 with level 43 and CSDME, 10 with level 35 and CSDME, and 1 with level 14 and CSDME. Further analysis of all the false-negatives showed that 2 centers accounted for 90 of 134 false-negatives (67%) with 21 of 90 (23%) having vtDR. Thus, for the other 5 nonretina centers, the rate of vtDR among the false-negatives was 5 (11%) of 44.

The false-positive rate was higher for the EyeArt AI system (90/792, 11%) than for the ophthalmologists (3/792, 0.3%). The EyeArt AI false-positives were comprised mostly of eyes with mild NPDR (44 eyes) and 17 other diagnoses, including drusen, nevi, epiretinal membrane, retinal vein occlusion, geographic atrophy, asteroid hyalosis, optic nerve swelling, peripapillary atrophy, uveitis, retinal pigment epithelium disturbance, and fibrous tissue (Fig 2 C–E). For the ophthalmoscopy, the false-positive diagnoses per Reading Center were no apparent DR (2 eyes) for retina specialists and chorioretinal scars for general ophthalmologists (1 eye).

## Discussion

In a point-of-care screening prospective study, the EyeArt AI system's sensitivity for detection of mtmDR was much higher than either a general ophthalmologist or retina specialist as compared with the clinical reference standard of grading of fundus photographs by a central Reading Center. Furthermore, the AI system, similar to the retina specialist group, did not miss any cases of vtDR, in contrast to the general ophthalmologist group. Although the AI system had a higher rate of false-positives than either a general ophthalmologist or retina specialist, these were, in general, eyes with mild NPDR or that had other ocular pathology that, in the community, would benefit from being referred and evaluated by an ophthalmologist. The demographic diversity, geographic diversity, and the wide spectrum of DR severity included in this study population support the generalizability of this study’s results to the intended DR screening population.

The significant discrepancy between the clinical reference standard grading and the retina specialists is mainly due to the undergrading of moderate NPDR as mild NPDR. None of the eyes had a severe NPDR grade or higher. It is well known that color fundus photographs result in higher DR grades than ophthalmoscopic examination. Scanlon et al^11^ explain that the difference in referable retinopathy detection between ophthalmoscopy and 7-field stereophotography was primarily because of difficulty in distinguishing hemorrhages and microaneurysms. ETDRS grading based on 4-widefield (or 7 field) stereo photographs is considered to be the clinical reference standard for DR diagnosis, including by the FDA for this study and other comparison studies. An AI screening-based system is ideally one that has high sensitivity so as not to miss the significant pathology that could cause visual loss. Conversely, an ideal AI screening system would have a lower specificity in the trade-off between sensitivity and specificity when screening for potentially visual threatening pathology. The EyeArt system fulfills these requirements and is a potentially useful screening tool with its high sensitivity for detecting mtmDR. Compared with dilated ophthalmoscopy for DR screening, advantages include the low cost and the convenient location as a point-of-care tool. Unlike human-based telescreening systems, the EyeArt system provides immediate determination of the presence of mtmDR that is available to the patient before leaving the primary care office, which has been shown to improve adherence to follow-up care.^16^ A referral can then be made, and the importance of follow-up can be stressed to the patient.

Disadvantages include the somewhat lower specificity that may lead to overreferral, although a significant portion of false-positives (i.e., overreferrals) had mild NPDR or other ocular pathology that would benefit from the evaluation by an ophthalmologist. In addition, patients who do not have mtmDR may not be referred for an ophthalmic examination that could screen for other ocular diseases. Other ocular conditions, such as refractive errors, glaucoma, and cataract, are not addressed in these individuals. Thus, use of the system should be tempered with advice to seek a general ophthalmic examination if the patient has blurred vision or if the patient is in a high-risk group for conditions such as glaucoma.

Given the current low rate of compliance with the recommendation for an annual diabetic retina examination, this system can be a useful adjunct in the detection of mtmDR and seems to be more accurate than clinical ophthalmoscopy for routine retinal screening.
